# P16/Ki-67 Immunostaining is Useful in Stratification of Atypical Metaplastic Epithelium of the Cervix

**DOI:** 10.4137/cpath.s522

**Published:** 2008-03-19

**Authors:** Ann E. Walts, Shikha Bose

**Affiliations:** Department of Pathology and Laboratory Medicine, Cedars-Sinai Medical Center, Los Angeles, California 90048

**Keywords:** P16, Ki-67, cervical intraepithelial neoplasia (CIN), human papilloma virus (HPV), atypical squamous metaplasia

## Abstract

Cervical metaplastic squamous epithelium exhibiting atypia insufficient for a diagnosis of cervical intraepithelial neoplasia (CIN) is usually reported as “atypical squamous metaplasia” (ASM). Stratification impacts treatment since the differential is often between reactive and high grade CIN (CIN II, III). Diagnosis with H&E is associated with low intra/interobserver concurrence. P16/Ki-67 immunostains are helpful to assess cervical biopsies for HPV-associated lesions but staining in metaplastic squamous epithelium has received little attention. This study aims to establish staining characteristics of metaplastic squamous epithelium and determine if p16/Ki-67 is useful in ASM stratification. 80 cervical biopsies containing morphologically normal and dysplastic squamous metaplasia were retrieved to determine the staining characteristics of metaplastic epithelium utilizing p16/Ki-67 immunostains. These included 21 benign squamous metaplasia (BSM) from benign cervices, 15 BSM present adjacent to HPV/CIN lesions, and 44 CIN involving squamous metaplasia. Serial sections with controls were stained for p16 and Ki-67 and in-situ hybridization (ISH) for low-risk (LR) and high-risk (HR) HPV was performed. P16 was recorded as negative, spotty, or band-like. Ki-67 was recorded as positive when present in >50% of lesional nuclei. Results were correlated with H&E diagnosis. 95% of the BSMs, whether from normal cervices or adjacent to HPV/CIN were p16/Ki-67 negative. 81% HG CINs involving squamous metaplasia were p16 band/Ki-67 positive. Low grade CIN (CIN I) involving metaplastic epithelium showed a broad distribution of p16/Ki-67 staining patterns. Based on these criteria, 20 ASM were evaluated. 10% of the ASM cases were p16 band/Ki-67 positive indicating HG CIN. 60% of the ASMs were p16/Ki-67 negative indicating reactive change (all with the exception of one case being HPV negative). The remaining 30% of the ASM cases showed variable positivity for p16 and Ki-67 and could not be stratified into the two categories. Thus p16/Ki-67 staining is helpful in stratification of ASM as reactive or CIN.

## Introduction

Cervical metaplastic squamous epithelium exhibiting atypia insufficient for a diagnosis of cervical intraepithelial neoplasia (CIN) is usually reported as “atypical squamous metaplasia” (ASM). Stratification of ASM into reactive and CIN groups impacts treatment since the differential is often between reactive and high (rather than low) grade CIN. Small biopsies, suboptimal orientation of sections, coexistent inflammatory/reactive cellular changes, and subjective criteria contribute to low levels of intra- and inter-observer concurrence when the diagnosis of ASM is based on H&E stains alone. Adjuncts that could help stratify ASM would be helpful in patient management.

It has been shown that p16/Ki-67 immunostains are helpful in the assessment of cervical (Sano et al. 1998; Keating et al. 2001; Klaes et al. 2001; Agoff et al. 2003; Hu et al. 2005; O’Neill and McCluggage, 2006) and anal (Walts et al. 2006) biopsies for human papillomavirus (HPV)-associated lesions but staining in squamous metaplasia has received little attention to date (Keating et al. 2001; Duggan et al. 2006; Iaconis et al. 2007; Kong et al. 2007; Regauer and Rich, 2007). Briefly, p16 is a cyclin-dependent kinase inhibitor that regulates transition from the G1 to the S phase of the cell cycle (Ortega et al. 2002). P16 has been shown to be upregulated and overexpressed in most high grade cervical dysplasias and carcinomas induced by high-risk (HR) HPV subtypes (Sano et al. 1998; Klaes et al. 2001). Ki-67 is a cell proliferation marker that is expressed during all phases of the cell cycle except G0 (Indinnimeo et al. 2000). This study was designed to establish baseline staining characteristics in squamous metaplastic epithelium so as to determine if p16/Ki-67 immunostaining is useful in stratification of ASM.

## Materials and Methods

The study was performed in two parts. The first part was designed to determine the p16/Ki-67 staining characteristics of morphologically normal and dysplastic metaplastic epithelium so as to develop algorithms for application to ASM. In the second portion of the study these algorithms were tested on a set of consecutive cases that had been reported as ASM in order to determine if it were possible to stratify cases of ASM into dysplastic or reactive groups.

After IRB approval, slides of 80 formalin fixed paraffin embedded cervical biopsies were retrieved from our files and divided into three groups based on examination of routine H&E stained sections: (i) Benign squamous metaplasia (BSM) without morphologic evidence of HPV infection or CIN obtained from benign cervices (21 cases) (ii) uninvolved (benign) squamous metaplasia adjacent to areas with evidence of HPV infection or CIN (15 cases) (iii) metaplastic epithelium involved by HPV or CIN (44 cases). All diagnoses had been rendered by experienced gynecologic pathologists and all were confirmed by the authors (AW, SB) on slide review. Cases with discrepant diagnoses were excluded from the study. No additional exclusion criteria were applied in case selection. For the second portion of the study 20 consecutive cases of ASM were retrieved from the files. These cases had also been reported by the same experienced gynecologic pathologists. An additional 10 ectocervical biopsies with HPV and/or CIN were evaluated as controls. The specimens had been collected from 84 women ranging in age from 10 to 71 years (mean 36.6 yrs; median 32.5 yrs).

Serial sections of the retrieved slides and appropriate controls were immunostained for p16 (Clone JC8 at dilution of 1:200 from Biocare Medical, Concord CA) utilizing Envision Plus Mouse Detection (DAKO, Carpinteria CA) and a DAKO Autostainer and for Ki-67 (CONFIRM Anti-Ki67 (k-2) primary antibody from and as diluted by Ventana Medical Systems, Inc. Tucson, AZ). As positive controls for p16 and Ki-67 immunostains we utilized paraffin sections of a HR HPV positive squamous carcinoma and of a tonsil, respectively. Sections of the cervical biopsies run substituting mouse IgG for p16 and Ki-67 antibody served as negative controls. Slides were treated with 3% hydrogen peroxide to block endogenous staining and the Envision polymer-based system was utilized for antigen detection. P16 antigen retrieval was accomplished by 30 minute incubation with steam and low pH buffer and Ki-67 antigen retrieval was accomplished by pretreatment on the Ventana Benchmark using CC1 buffer. The chromagen for both antibodies was DAB (3,3’-diaminobenzidene). P16 was recorded as negative (staining in 0%–25% of lesional cells), spotty (positive in >25% of cells distributed throughout the lesion, or band-like (positive in >90% of contiguous lesional cells). Nuclear and/or both nuclear and cytoplasmic staining was interpreted as positive for p16; cytoplasmic staining alone was considered nonspecific and recorded as negative. Ki-67 was recorded as positive when present in >50% of lesional cell nuclei. Positive staining for Ki-67 was exclusively nuclear.

Serial sections of 88 (88%) of the biopsies (14 BSM, 12 BSM adjacent to CIN, 43 CIN in squamous metaplasia, 19 ASM) and 10 (100%) of the ectocervical controls were subjected to in-situ hybridization (ISH) for high risk (HR) HPV utilizing HPV-III Family 16 (Ventana). Serial sections of 93 (93%) of the biopsies (15 BSM, 15 BSM adjacent to CIN, 44 CIN in squamous metaplasia, 19 ASM and 10 (100%) of the ectocervical controls were also subjected to in-situ hybridization for low risk (LR) HPV utilizing Inform HPV II-Family 6 and as per the manufacturer’s recommendations. Presence of LR and HR HPV was determined without knowledge of the p16/Ki-67 staining.

Immunostaining, ISH, and H&E diagnoses were correlated. Statistical analyses were performed using the Fisher exact test. A 2-sided p value of <0.05 was considered significant.

Followup was available for 10 (50%) of the ASMs up to 19 months after the index samples had been obtained.

## Results

BSM from morphologically normal cervices and BSM adjacent to HPV/CIN showed similar staining and were negative for p16 and Ki-67 (Table 1). Only two BSMs from morphologically normal cervices showed spotty p16 staining in 25%–50% of metaplastic cells and both of these were Ki-67/HR and LR HPV negative. HPV (LR and/or HR) ISH was negative in all of the 33 (100%) BSMs that were tested.

CINs involving metaplastic epithelium showed staining similar to CIN in ectocervical epithelium. Band-like p16 immunostaining and Ki-67 positivity were present in 22 of 27 (81.5%) HG CINs involving metaplastic epithelium. Four of the remaining 5 HG CINs involving metaplastic epithelium showed p16 positivity (2 band and 2 spotty >75%) of which 2 were Ki-67/HR HPV DNA positive, one was Ki-67/HPV DNA negative, and one was Ki-67 negative/HR HPV DNA positive. The fifth case (reported as CIN II) was p16/Ki-67/HPV negative. LG CIN involving metaplastic epithelium showed a broad distribution of p16/Ki-67 immunostaining patterns ranging from negative to positive.

Of the 10 ectocervical control cases, four were benign reactive ectocervical biopsies and all were p16/Ki-67/HPV negative, two were HG CIN and were p16 band/Ki-67 positive, one of which was also HR HPV ISH positive. Four were LG CIN and showed variable staining for p16 and Ki-67; two were positive for LR HPV, one was positive for HR HPV, and one was positive for both LR and HR HPV by ISH.

Thus to summarize, irrespective of the presence of adjacent HPV/CIN, morphologically normal and dysplastic lesions in metaplastic epithelium show a similar staining pattern to that seen in normal and dysplastic ectocervical epithelium, respectively. HG CIN is p16 band/Ki-67 positive, LG CIN shows a variable staining pattern, and benign squamous/metaplastic epithelium is p16/Ki-67 negative.

**Figures 1 and 2.**
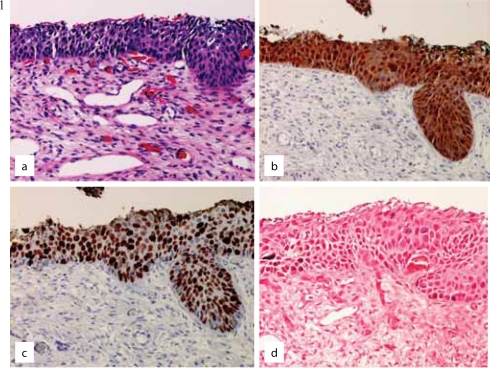

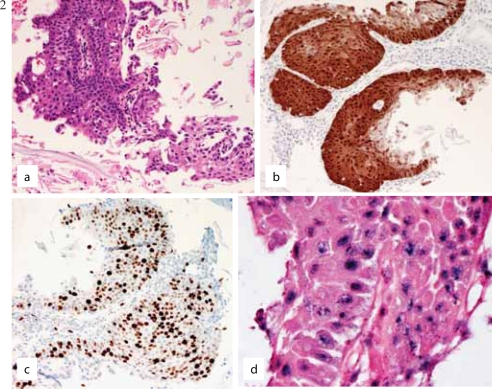
(**a**) Atypical squamous metaplasia with (**b**) band-like p16 staining, (**c**) positive Ki-67, and (**d**) positive HR HPV on ISH consistent with HG CIN.

**Figure 3.**
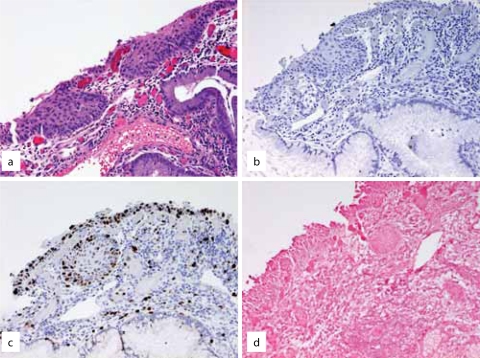
(**a**) Atypical squamous metaplasia negative for (**b**) p16, (**c**) Ki-67, and (**d**) HPV consistent with reactive change.

Based on the above staining patterns, cases with ASM could be further stratified into CIN and benign groups (Figs. 1–3). Of the 20 ASMs, 5 were p16 band positive; of these 4 were HR HPV positive while only 2 were Ki-67 positive. Fourteen (70%) ASMs were p16 negative; of these 13 were HPV negative with 11 also being Ki-67 negative. One ASM showed spotty p16 staining in 25%–50% of lesional cells; this biopsy was Ki-67 negative but HPV ISH was not done. Only one of the ASMs that were p16/Ki-67 negative was HR HPV ISH positive. Thus 14 (70%) of the ASMs (Table 1) could be reclassified, 2 (10%) as HG CIN and 12 (60%) as reactive changes.

Table 2 summarizes the p16 staining patterns in relation to H&E diagnosis and HPV ISH. Of the 33 p16 band-positive cases, 25 (76%) were HR HPV positive while 8 cases were negative. These included 5 HG CIN cases with positive Ki-67 and 1 LG CIN, 1 ASM, and 1 HG CIN each with negative Ki-67. All of the three cases with spotty p16 positivity in >75% and Ki-67 positivity in >50% of lesional cells were associated with HR HPV subtypes. P16 staining in <25% of cells was strongly associated with HPV negativity by ISH (p < 0.0001).

Table 3 summarizes Ki-67 positivity in >50% of lesional cells in relation to H&E diagnosis and HPV ISH. Of the 34 Ki-67 positive cases, 27 (79.4%) were HR HPV positive while 7 cases (2 ASMs and 5 HG CINs) were HR HPV negative.

Followup (range: 1 to 19 months; mean: 7.4 months; median: 6.0 months) for 50% (10) of the ASMs is shown in Table 4. Six of 8 cases that were p16/Ki-67 negative were negative on followup including one case that was HR HPV positive. One case that was p16/Ki-67/HR HPV negative showed LG CIN on followup and one case that was p16/Ki-67 negative showed ASM favored to be reactive on followup. Both of the p16 positive ASM cases that had followup were also HR HPV positive. One was Ki-67 positive, had HG CIN in a prior and concurrent biopsy, and ASCUS on followup while the other was Ki-67 negative, had LG CIN on a prior biopsy, and HG CIN on followup.

## Discussion

In this study we have shown that p16/Ki-67 Immunostaining is useful in stratification of ASM into reactive and CIN groups by using the following criteria: (i) p16/Ki-67 negativity is indicative of a reactive change, (ii) band-like staining for p16 and Ki-67 positivity in >50% of lesional nuclei is indicative of HG CIN and has a high correlation with HPV ISH positivity, (iii) p16 band positivity or spotty positivity in >75% of lesional cells by itself is suggestive of HPV infection, (iv) Ki-67 positive staining in >50% of nuclei with negative or spotty staining for p16 in <25% of lesional cells remains a nonspecific finding often observed in reactive epithelium. Our findings are concordant with p16/Ki-67 immunostaining reported by others in cervical biopsies (Sano et al. 1998; Keating et al. 2001; Klaes et al. 2001; Agoff et al. 2003; Hu et al. 2005; Duggan et al. 2006; O’Neill and McCluggage, 2006; Iaconis et al. 2007; Kong et al. 2007; Regauer and Rich, 2007) and by us in anal biopsies (Walts et al. 2006) and indicate that observations made in these epithelia can be applied to ASM. Seventy per cent of our cases with ASM could be reclassified into reactive group or CIN. The remaining 30% showed equivocal staining results of which a third showed evidence of HPV infection by ISH.

Human papillomavirus (HPV) is the most common sexually transmitted infection in the United States with as many as 20 million persons infected (Torpy et al. 2007). The overall prevalence of infection is estimated at 26.8% in females 14 to 59 years of age with the prevalence of high and low-risk HPV subtypes estimated at 15.2% and 17.8%, respectively (Dunne et al. 2007). While HPV infection is very common, it is believed that up to 90% of infections clear spontaneously within 2 years (Moscicki et al. 1998; Franco et al. 1999). Persistent infection by high-risk HPV subtypes has been shown to be the most consistent precursor to cervical carcinoma with HR HPV subtypes detectable in as many as 99% of cervical carcinomas (Walboomers et al. 1999).

Squamous metaplasia of the cervix is so common that it is virtually regarded as “normal”. Squamous metaplasia can be unifocal but is more frequently multifocal and almost always involves the transformation zone which is also involved in most CINs. Hence it is not surprising that ASM is a frequent diagnosis in surgical pathology. Often a tissue diagnosis of ASM is preceded by a cytological diagnosis of ASC-H (atypical squamous cells of undetermined significance, cannot exclude high grade squamous intraepithelial lesion) (Solomon et al. 2002) which according to current clinical guidelines is an indication for colposcopy and biopsy without reflex HPV (Wright et al. 2002) thus contributing to the expectation that biopsy will provide a definitive diagnosis upon which appropriate patient management can be based. While a biopsy diagnosis of CIN in metaplastic epithelium is an indication for cervical LEEP (loop electrosurgical excision procedure) or conization and a diagnosis of squamous metaplasia with reactive cellular changes would not elicit further evaluation, a diagnosis of ASM not otherwise specified presents gynecologists with a dilemma in patient management. Because the difference in management is great, there is a need for pathologists to be able to reliably stratify ASM as reactive or CIN and to minimize the use of ASM as a diagnosis. Our findings indicate that p16/Ki-67 immunostains can be applied toward this end.

In our study cohort, the majority of biopsies that had been reported as ASM were p16/HPV negative suggesting that the atypia in these epithelia is reactive rather than CIN. Review of the literature indicates that regardless of whether immunostains, ISH, PCR, or followup is used as the gold standard for HPV, the tendency to “overdiagnose” squamous metaplasia as ASM is not unique to our laboratory. In a study utilizing CK17, p16, and p63 immunostains to distinguish atypical immature squamous metaplasia from HG CIN, Regauer (Regauer and Rich, 2007) reported that 59% (10 of the 17 cases that were reclassified as benign. In another study of cervical biopsies utilizing clinical followup data, Duggan (Duggan et al. 2006) found that among 32 ASMs for which they had followup, the followup was benign in 34.4% of cases and among the 21 that developed CIN, only 5 (24%) were HG CINs. Kong (Kong et al. 2007) in a study utilizing PCR found that 14 (53.8%) of 26 biopsies reported as ASM were negative for HPV by PCR while in another study Keating (Keating et al. 2001) found that of 12 ASMs, 50% were negative for HPV by PCR. While it should be acknowledged that each of these methodologies has its limitations as the “gold standard” for CIN, these reports attest to the necessity for pathologists to hone the use of ASM as a diagnosis.

To date, we are aware of few other studies (Keating et al. 2001; Duggan et al. 2006; Iaconis et al. 2007; Kong et al. 2007; Regauer and Rich, 2007) that have specifically addressed p16/Ki-67 immunostaining in ASM of the cervix. Differences in case selection, antibody clones, staining conditions, definitions of “positive” immunostaining, biomarkers studied separately and in combination with p16 and/or Ki-67, and use of PCR vs. ISH as the “gold standard” for HPV, limit comparisons between these studies. In general, all workers found that overexpression of p16 (be it “band-like” staining for us or “diffuse strong positivity” for others) is strongly associated with HR HPV positivity (whether assayed by ISH or PCR) while focal or negative p16 staining is characteristic of LG CIN or reactive atypia. Duggan (Duggan et al. 2006) concluded that “p16 positivity and Ki-67 high index correlates with HG CIN and … the combination is useful in stratification of some ASMs as HG CIN”. Iaconis (Iaconis et al.) also reported that the combination of p16 and Ki-67 immunostains is helpful in decreasing the number of atypical immature squamous metaplasia cases with “nondiagnostic atypia”. Keating (Keating et al. 2001) noted that Ki-67 positivity can be seen in the upper two-thirds of BSM epithelium that is tangentially sectioned and/or inflamed and that Ki-67 by itself has a low specificity as a biomarker for HPV. They also noted that “the association of p16 staining and HR HPV was not absolute” and suggest that not all HPV subtypes currently classified as “HR” have the same ability for p16 upregulation. In their study using PCR, they comment that “HPV DNA sequences are not always identified/confirmed in histological sections” that showed a “morphologic abnormality”. In this regard, it should be noted that a negative HPV ISH can occur in the presence of an HPV subtype that was not tested for (ISH probes we utilized only test for two LR and 12 HR subtypes) or when the HPV DNA present is below the detection threshold of the assay employed. Each study acknowledges the limitations in sensitivity and specificity of any single biomarker and emphasizes the benefit of utilizing a combination of biomarkers for optimal resolution of problematic biopsies. In our study, addition of Ki-67 positivity in >50% of lesional cells to p16 band positivity as diagnostic requisites increased the specificity (from 92.5% to 96%) and the positive predictive value (from 85.7% to 91.7%) for a diagnosis of HG CIN (Table 5).

## Figures and Tables

**Table 1.** P16, Ki-67, and HPV staining in squamous metaplastic epithelium (N = 100).

**p16**	**0%–25%**	**0%–25%**	**25%–75%**	**25%–75%**	**>75%**	**>75%**	**Band**	**Band**
**Ki-67**	**<50%**	**>50%**	**<50%**	**>50%**	**<50%**	**>50%**	**<50%**	**>50%**
BSM in morphologically normal cervices (21 cases)								
HPV–	9	0	2	0	0	0	0	0
LR–/HRND	4	0	0	0	0	0	0	0
HR–/LRND	3	0	0	0	0	0	0	0
HPV ND	3	0	0	0	0	0	0	0
BSM adjacent to CIN (15 cases)								
HPV–	12	0	0	0	0	0	0	0
LR–/HRND	3	0	0	0	0	0	0	0
CIN in Metaplasia (44 cases)								
LG CIN (17)								
HPV–	4	0	1	0	0	0	1	0
LR+/HRND	1	0	0	0	0	0	0	0
LR–/HR+	3	2	0	1	0	1	1	2
HG CIN (27 cases)								
HPV–	1	0	0	0	0	0	1	5
LR–/HR+	0	0	0	0	0	2	1	17
ASM (20 cases)								
HPV–	11	2	0	0	0	0	1	0
LR–/HR+	1	0	0	0	0	0	2	2
HPV ND	0	0	1	0	0	0	0	0

**Abbreviations:** LR–, ISH negative for LR HPV; HR–, ISH negative for HR HPV; LR+, ISH positive for LR HPV; HR+, ISH positive for HR HPV; LRND, ISH for LR HPV not done; HRND, ISH for HR HPV not done; HPV ND, ISH for HPV not done.

**Table 2A.** P16 immunoreactivity and diagnosis (N = 100).

**p16**	**Total**	**HG CIN**	**LG CIN**	**Negative**	**ASM**
Band	33	24	4	0	5
S > 75%	3	2	1	0	0
S25%–75%	5	0	2	2	1
S < 25%	59	1	10	34	14

**Table 2B.** P16 immunoreactivity and HR HPV by ISH (N = 100).

**p16**	**Total**	**HR+**	**HR−**	**HR ND**
Band	33	25	8	0
S > 75%	3	3	0	0
S25%–75%	5	1	3	1
S0%–25%	59	6	42	11

**Table 3A.** Ki-67 immunoreactivity and diagnosis (N = 100).

**Ki-67**	**Total**	**HG CIN**	**LG CIN**	**Negative**	**ASM**
Positive (>50%)	34	24	6	0	4
Negative (<50%)	66	3	11	36	16

**Table 3B.** Ki-67 immunoreactivity and HR HPV by ISH (N = 100).

**Ki-67**	**Total**	**HR+**	**HR−**	**HR ND**
Positive (>50%)	34	27	7	0
Negative (<50%)	66	8	46	12

**Table 4.** Followup for ASMs (N = 10).

**Initial profile**	**Followup**
P16+/Ki–67+/HR+	Paps @ 5 and 14 months: ASCUS
P16+/Ki–67–/HR+	Cone @ 6 months: HG CIN
P16–/Ki–67–/HR ND	LEEP @ 1 month: focal ASM (favor reactive)
P16–/Ki–67–/HR–	Paps @ 3,7,11,15, and 19 months: all negative
P16–/Ki–67–/HR–	Hysterectomy @ 5 months: negative
P16–/Ki–67–/HR–	Pap @ 6 months: negative
P16–/Ki–67–/HR–	Hysterectomy @ 8 months: negative
P16–/Ki–67–/HR–	Cone @ 6 months: LG CIN
P16–/Ki–67–/HR+	Pap @ 7 months: negative
P16–/Ki–67–/HR–	Hysterectomy @ 2 months: negative

**Table 5.** Sensitivity, specificity, positive and negative predictive value for HG CIN (N = 80; ASMs omitted).

	**Sensit**	**Spec**	**PPV**	**NPV**
p16 band	89	93	86	94
Ki-67 > 50%	89	89	80	94
p16 band and Ki-67 > 50%	81	96	92	91
